# Longitudinal associations between socioeconomic status and psychosocial problems in preschool children

**DOI:** 10.1007/s00787-023-02217-5

**Published:** 2023-05-17

**Authors:** Jie Luo, Amy van Grieken, Ingrid Kruizinga, Hein Raat

**Affiliations:** Department of Public Health, Erasmus Medical Center, Rotterdam, The Netherlands

**Keywords:** Socioeconomic status, Mental health, Preschool children, Neighborhood socioeconomic status

## Abstract

Socioeconomic status (SES) at different points in a child’s lifetime may have different effects on health outcomes. This study aimed to examine longitudinal associations between SES and psychosocial problems in preschool children (*n* = 2509, *M*_age_ = 24.2 ± 1.3 months). The psychosocial problems of children were assessed using the Brief Infant–Toddler Social and Emotional Assessment at age 2 years and age 3 years and categorized as having yes/no psychosocial problems. Four groups of pattern of presence/absence of psychosocial problems between age 2 and 3 years were classified: (1) ‘no problems’, (2) ‘problems at age two’, (3) ‘problems at age three’, and (4) ‘continuing problems’. Five indicators of SES (i.e., maternal education level, single-parent family, unemployment, financial problems, and neighborhood SES) were evaluated. Results showed around one-fifth (2*Y* = 20.0%, 3*Y* = 16.0%) of children had psychosocial problems. Multinomial logistic regression models revealed low and middle maternal education levels were associated with ‘problems at age two’; low maternal education level and financial problems were associated with ‘problems at age three’; low and middle maternal education level, single-parent family, and unemployment were associated with ‘continuing problems’. No associations were observed between neighborhood SES and any pattern. Results suggest children in a lower SES, indicated by maternal education, single-parent family, and financial stress, had higher odds of developing and continuously having psychosocial problems in early childhood. These findings call for optimally timing interventions to reduce the impact of disadvantaged SES in early childhood on psychosocial health.

**Supplementary Information:**

The online version contains supplementary material available at 10.1007/s00787-023-02217-5.

## Introduction

Psychosocial health and well-being in childhood concern child’s ability to perform well at school and socially, with a general impact on their overall well-being [1]. Problems in psychosocial health and well-being can concern issues with regard to regularizing emotions, behavior, and social interactions [2]. According to symptoms, psychosocial problems in children and adolescents can broadly be divided into three categories: emotional/internalizing problems (e.g., depressive symptoms; anxiety), behavioral/externalizing problems (e.g., oppositional defiant; hyperactivity), and social problems (e.g., difficulties in social activities with peers) [2]. Approximately 13.4% of children aged 6–18 years worldwide have psychosocial problems, according to the above definition of problems in depressive symptoms, anxiety, attention-deficit hyperactivity, oppositional defiant, disruptive disorder, and conduct disorder [3]. Among children 2–6 years, problems are reported by Charach et al. in 17.6% of children [4]. The prevalence of psychosocial problems in children and adolescents is rising [4–6]. It is known that problems in early childhood can track into adulthood [1, 7], and therefore, early detection of (risk for) psychosocial problems is needed to provide timely support to children families [5]. In addition, insight into the absence and presence of psychosocial problems over time in young children are relevant for researchers and professionals to be able to further personalize support offered.

Socioeconomic characteristics of families are known to influence childhood health and well-being [8, 9]. Socioeconomic status (SES) can be referred to as a combination of individual and contextual socioeconomic factors [9]. Potential factors that impact socioeconomic status include social inequality [10], the health care system [11, 12], and culture [13]. The impact of factors varies between countries. For example, a global study showed that children from countries with low- and middle-income and education suffered from diseases much more frequently than peers from countries with high income and education [14]. Moreover, the former children with psychosocial diseases had fewer opportunities to be diagnosed and treated likewise [15, 16]. Therefore, how SES might affect psychosocial problems should be studied within a certain context. In the Netherlands, research has shown that family SES (i.e., the individual-level indicator based on parental education level, employment, and family income) and neighborhood SES (i.e., the area-level indicator based on poverty rate, unemployment rate, and adults’ education level) were associated with psychosocial health of children and adolescents aged 4–17 years measured with Strengths and Difficulties Questionnaire (SDQ) [17–19]. Weinberg et al. studied only parental and adolescent-perceived SES [19]; Brons et al. focused on the neighborhood SES together with school SES [18]; and Boelens et al. studied the independent contribution of family SES and neighborhood SES to children and adolescents’ psychosocial health [17]. Therefore, studying both family SES and neighborhood SES could provide comprehensive information to understand and conduct intervention projects to improve children’s psychosocial health [20].

The SES and health relation across the lifespan could be dynamic and vary in domains across different life stages [21, 39]. For example, associations of SES with global health measures are similar across childhood and adolescence, whereas associations of SES with specific acute conditions vary by age among a sample of nationally representative US children [21]. Research on SES and psychosocial problems is mostly conducted in school-age children and adolescents in the Netherlands and other European countries, while evidence on associations between SES and psychosocial problems in preschool children (≤ 4 years) is relatively scarce [17–19, 22, 23]. Moreover, studies on longitudinal relation between SES and psychosocial problems are needed for long-term intervention projects.

The present study’s first aim was to describe the presence and absence of psychosocial problems in preschool children at age 2 and 3 years and define four patterns of psychosocial problems (i.e., no problems, problems at age 2, problems at age 3, and continuing problems). The second aim was to study the associations between indicators of SES, namely maternal education level, single-parent family (yes/no), parent unemployment in the family (yes/no), financial problems in the family (yes/no), and neighborhood SES, with these patterns of psychosocial problems. We hypothesized that relatively low-SES at age 2 years would be associated with the child having (continuing) psychosocial problems at age 2 and 3 years.

## Methods

### Ethics statement

Parents received written information about the study and were free to refuse to participate or stop participation at any time. The Medical Ethical Committee of the Erasmus Medical Center Rotterdam gave the permission to carry out the study (MEC-2014-152; MEC-2009-092). This study was conducted following the guidelines proposed in the World Medical Association Declaration of Helsinki. Consent forms were obtained from participants. Data used in this study were anonymous on the work platforms.

### Study design and population

Participants included in the present study from two cohorts concerning child psychosocial health. One cohort study (cohort 1) evaluated the early detection tool for psychosocial problems in toddlers: Brief Infant–Toddler Social and Emotional Assessment (BITSEA) [24]. Another cohort (cohort 2) studied questionnaire pressure when identifying psychosocial problems. More details can be found elsewhere [25]. The data from these two observational cohorts were combined to enlarge the sample size in this study. The combined population in this study was recruited similarly in Rotterdam–Rijnmond area. Every family with a 2-year-old child in this area was invited by letter to participate weeks before the well-child visit organized by YHC. The questionnaires used in the two cohorts were based on the regular questionnaire used in Youth Care Center (YHC). Data used in this study were collected from the common parts of two cohort questionnaires with the same questions. The two cohort studies were conducted in different years. Therefore, children participated only once in this study. The calculation of neighborhood SES used the data of the year when participants were recruited accordingly. The baseline questionnaire was sent to parents by mail a few weeks before the regular well-child visit at YHC at age 2 years. Parents could return the completed questionnaires during their visit. Parents of 3499 children completed the baseline questionnaire. One year later, of those parents who enrolled in the study, 2734 (78.1%) returned the completed questionnaire. For the information related to participants, we excluded second child of twins by the order of inclusion (*n* = 26), other caregivers than parents (*n* = 32), and missing data on the BITSEA at baseline or follow-up (*n* = 64). Due to delay in delivery or response, only a few parents (*n* = 60) sent the follow-up questionnaires for 3-year-old children back when the child was already over 42 months (i.e., 3.5 years) old. These data were removed because of the age limit of BITSEA (12–36 months). The upper age limit was modified to 42 months, because differences in psychosocial development between children age 3 years and 3.5 years did not show a significant difference in the previous research. Thus, 2509 participants were included in the analyses of this study (see Supplementary Fig. 1).

### Measurements

#### Socioeconomic status

Socioeconomic status in the present study includes family SES, and neighborhood SES measured at age 2. Family SES was measured by maternal education level, single-parent family (yes/no), parent unemployment in the family (yes/no), and financial problems in the family (yes/no). Parental education level was the highest level of education finalized by the respondent and their partner and categorized as high, middle, or low following the Classification of Statistics Netherlands [26]. Unemployment and financial problems in the family were derived from 12 stressful stress events based on Tiet et al.’s [25] Adverse Life Events Scale. Participants were asked whether unemployment happened in the family in the past 2 years when child age 2 years. Financial problems were assessed in the same measurement.

Neighborhood SES in the present study was assessed at baseline. Data to calculate neighborhood SES were obtained from the Netherlands Institute of Social Research (SCP) (Netherlands Institute of Social Sciences, 2019). The SCP computes a neighborhood SES score based on mean adult income, percentage of low adult incomes, percentage of low-educated adult residents, and percentage of unemployed adult residents in a neighborhood using principal component analysis. By matching the number of postal codes of the neighborhood, socioeconomic status scores of the 213 neighborhoods participating in the study were trisected to create low-, middle-, and high neighborhood SES groups.

#### Psychosocial problems

Among preschool children, psychosocial problems are displayed externally as social–emotional or behavioral problems (e.g., depressive emotions and hypertension) parallel to delays in social-emotional or behavioral competence (e.g., indifference and noncompliance) [27]. Psychosocial problems of the children in this study were measured by BITSEA, a reliable and validated instrument [24]. The BITSEA consists of 41 items, and each item is scored 0 for ‘not true’, 1 for ‘somewhat true’, and 2 for ‘certainly true’ [28]. The BITSEA is comprised of two scales, a 31-item Problem scale and an 11-item Competence scale which measures social-emotional problems and delay in social–emotional competence of children 12–36 months. The items from the two scales of BITSEA are summed up independently. A score ≥ 14 on the Problem scale was categorized as ‘at risk of psychosocial problems’, and a score ≤ 15 on the competence scale was categorized as ‘at risk of competence delay’ [24, 29]. The child who scored at-risk on either Problem scale, Competence scale, or both was regarded as having psychosocial problems. The Cronbach’s alphas in this study are 0.73 and 0.63.

Psychosocial problems were assessed at two time points: the 24-month well-child visit and (2*Y*) and the 36-month well-child visit (3*Y*). For this study, we classified the pattern of presence/absence of psychosocial problems between age 2 and 3 into four groups: (1) ‘no problems at age two and three’ (‘no problems’), (2) ‘problems at age two, but not at age three’ (‘problems at age two’), (3) ‘no problems at age two, but problems at age three’ (‘problems at age three’), and (4) ‘problems at age two and three’ (‘continuing problems’).

#### Other measurements

Sociodemographic characteristics of participants were assessed at age 2, including the respondent of baseline questionnaire, child age, child gender, child migration background, parental age, and parental migration background. Migration background (Dutch and migration) was defined based on the country of birth of both parents according to the Classification of Statistics Netherlands [26].

### Statistical analysis

Descriptive statistics were used to describe the general characteristics and SES of the study population. Significant differences between groups were accessed by independent samples *t* test or one-way analysis of variance for continuous variables and by Chi-square tests for categorical variables (*p* < 0.05).

Correlation analyses were conducted to examine the multicollinearity for indicators of SES. The maximal coefficient *r* = 0.508 between maternal education level and paternal education level indicated a medium correlation (0.4 < *r* < 0.6). Parental education level was excluded in multinomial regression models to decrease the multicollinearity. The rest were under 0.4; therefore, except parental educational level, maternal educational level, single-parent family, parent unemployment in the family, and financial problems in the family were added into the model.

The multinomial logistic regression model was fitted to investigate the longitudinal association between SES and the pattern of presence/absence of psychosocial problems. The model regarded indicators of SES as independent variables. Child gender and child migration background were added as covariates after checking correlations between potential covariates and psychosocial problems at age 2 (data not shown). ‘No problems’ group was regarded as the reference group in multinomial logistic regression among four groups of the pattern of presence/absence of psychosocial problems.

The binary logistic regression analyses were conducted to examine the associations between SES indicators and psychosocial problems in children at age 2 and age 3, respectively (Supplementary Table S1). Non-response analyses using Chi-square tests and *t* tests were conducted to compare sociodemographic characteristics of participants only in assessment at age 2 (*n* = 775) and participants in the assessment at age 3 (*n* = 2734) (supplementary Table S2).

Multiple imputation by fully conditional specification (FCS) was used to deal with the missing data on independent variables and covariates in SPSS [30]. Missing value analysis and missing value pattern were checked to apply the multiple imputations for the missing value in independent variables [27]. The pooled results of ten imputed datasets were used to report odds ratio (OR) and 95% confidence interval (CI). Finally, we performed a sensitivity analysis using complete-case data without missing values to check the robustness of the results (Supplementary Table S3).

The sociodemographic characteristics of the populations from the two cohorts were compared, and there were some significant differences (Supplementary Table S4). To take into consideration cohort 1 or cohort 2, a cohort variable (1 = cohort 1, 2 = cohort 2) was added to the logistic regression model (Supplementary Table S5). The association significances in the models kept still with or without the cohort variable. A *p* value < 0.05 was considered to be statistically significant. All analyses were completed using the IBM SPSS version 25 (IBM Corp., Armonk, NY, USA).

## Results

### Characteristics of participants

Table 1 shows the general characteristics and SES of the study population at age 2. The mean age of children was 24.2 (SD = 1.3) months, and 50.8% were girls. The mean age of parents was 33.7 (SD = 4.7) years, and most (90.1%) respondents of baseline questionnaires were mothers. Compared to children without psychosocial problems at age 2, children who had psychosocial problems were more likely to be younger, be boys, have a migration background, and have parents with a migration background (*p* values < 0.05). Regarding socioeconomic status, children who had psychosocial problems were more likely to have parents with low education levels, experience unemployment and financial problems in the family, and live in a lower SES neighborhood (*p* values < 0.05). When comparing the characteristics of the population in four groups of the pattern of presence/absence of psychosocial problems, each difference between groups was significant observed by the Chi-square tests (*p* values < 0.05).

**Table 1:** characteristics of participants in the analyses (*n* = 2509)

Items	Total (*n* = 2509)	Psychosocial problems at age 2		Pattern of presence/absence of social-emotional problems			
		No (*n* = 2007)	Yes (*n* = 502)	No problems (*n* = 1812)	Problems at age 2 (*n* = 296)	Problems at age 3 (*n* = 195)	Continuing problems (*n* = 206)
General characteristics							
Child age in months	24.2 ± 1.3	24.2 ± 1.3	24.0 ± 1.4	24.2 ± 1.6	23.9 ± 1.6	24.2 ± 1.3	24.2 ± 1.4
Mother age in years	33.7 ± 4.7	33.7 ± 4.6	33.8 ± 5.1	33.7 ± 4.6	34.1 ± 5.1	33.2 ± 5.2	33.3 ± 5.2
Father age in years	36.9 ± 5.4	36.9 ± 5.2	37.0 ± 6.2	37.0 ± 5.0	36.9 ± 5.5	36.8 ± 5.8	37.9 ± 7.4
Child gender							
Boy	1232 (49.2)	937 (46.8)	295 (58.8)	831 (46)	168 (56.8)	106 (54.4)	127 (61.7)
Child-ethnic background							
Migration	568 (23.1)	386 (19.6)	182 (37.5)	325 (18.2)	88 (30.9)	61 (32.8)	94 (47.0)
Respondent of baseline questionnaire							
Mother	2136 (90.1)	1730 (90.7)	406 (87.7)	1567 (90.8)	247 (90.5)	163 (89.1)	159 (83.7)
Maternal ethnic background							
Migration	647 (26.5)	440 (22.5)	207 (43.2)	377 (21.3)	111 (38.8)	63 (33.7)	96 (49.7)
Paternal ethnic background							
Migration	610 (25.0)	424 (21.6)	186 (38.8)	366 (20.6)	94 (32.8)	58 (30.9)	92 (47.7)
Family socioeconomic status							
Maternal education level							
High	1412 (57.8)	1207 (61.4)	205 (42.9)	1106 (62.1)	140 (49.3)	101 (54.3)	65 (33.5)
Middle	780 (31.9)	597 (30.4)	183 (38.3)	538 (30.2)	101 (35.6)	59 (31.7)	82 (42.3)
Low	253 (10.3)	163 (8.3)	90 (18.8)	137 (7.7)	43 (15.1)	26 (14)	47 (24.2)
Paternal education level							
High	1231 (52.1)	1052 (55.0)	179 (39.6)	964 (55.8)	126 (45.5)	88 (48.4)	53 (30.3)
Middle	810 (34.3)	639 (33.4)	171 (37.8)	573 (33.1)	97 (35)	66 (36.3)	74 (42.3)
Low	322 (13.6)	220 (11.5)	102 (22.6)	192 (11.1)	54 (19.5)	28 (15.4)	48 (27.4)
Single-parent family							
Yes	137 (5.6)	90 (4.6)	47 (9.9)	76 (4.3)	20 (7.1)	14 (7.6)	27 (14.0)
Unemployment in the family							
Yes	223 (9.1)	152 (7.7)	71 (14.4)	135 (7.6)	34 (11.6)	17 (8.8)	37 (18.5)
Financial problems in the family							
Yes	122 (5.0)	77 (3.9)	45 (9.2)	61 (3.4)	20 (6.8)	16 (8.2)	25 (12.7)
Neighborhood socioeconomic status							
High	927 (37.6)	777 (39.3)	150 (30.5)	709 (39.7)	102 (34.9)	68 (35.8)	48 (24.1)
Middle	429 (17.4)	354 (17.9)	75 (15.3)	329 (18.4)	47 (16.1)	25 (13.2)	28 (14.1)
Low	1112 (45.1)	846 (42.8)	266 (54.2)	749 (41.9)	143 (49)	97 (51.1)	123 (61.8)

Data presented as mean ± SD or number (percentage)

^a^Missing item: Child age = 136; Child gender = 5; Child-ethnic background = 55; Mother age = 185; Mother-ethnic background = 71; Mother-education level = 64; Father age = 1338; Father-ethnic background = 65; Father-education level = 146; Single-parent family = 83; Unemployment in the family = 46; Financial problems in the family = 49; Neighborhood socioeconomic status = 41

**Fig. 1:**
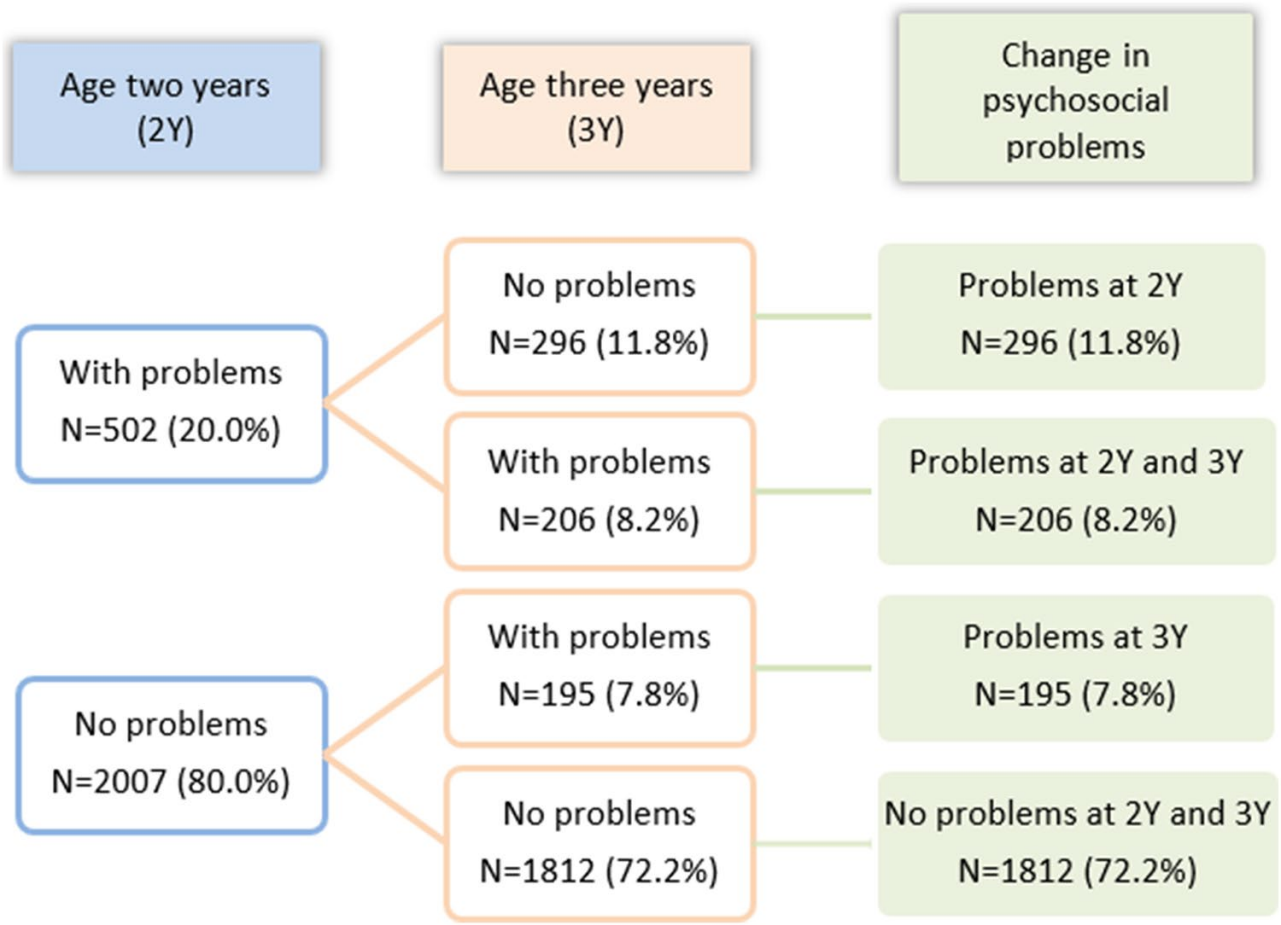
Frequency of psychosocial status of children

Figure 1 shows the frequency of children with psychosocial problems at age 2 and age 3 as well as pattern of presence/absence of psychosocial problems. First, 2007 (80%) children did not have psychosocial problems at age two, and 502 (20%) had psychosocial problems at age 2. After the assessment at age 3, a total of 72.2% of the sample continuously without psychosocial problems at both age 2 and 3 were in ‘no problems’ group; 8.2% continuously with psychosocial problems were in the ‘continuing problems’ group. The results of assessment of psychosocial problems for the rest children changed between age 2 and 3: 11.8% in ‘problems at age two’ group and 7.8% in ‘problems at age three’ group.

### Socioeconomic status and psychosocial problems

Supplementary Table S1 shows associations between SES indicators and psychosocial problems in children at age 2 and 3, respectively. The proportions of children having psychosocial problems were 20.0% at age 2 and 16.0% at age 3 assessed by BITSEA. The binary logistic regression analyses show that children of a mother with a low education level, experienced unemployment, and experienced financial problems in the family had higher odds of having problems compared to their peers at age 2. Children of a mother with a low education level, living in single-parent families, and experienced unemployment and financial problems in the family had higher odds of having problems at age 3 compared to their peers.

### Socioeconomic status and pattern of presence/absence of psychosocial problems

Table 2 presents the results of multinomial logistic regression models exploring the longitudinal association between SES indicators and pattern of presence/absence of psychosocial problems in children age 2–3 years.

**Table 2:** Multinomial logistic regression models (family socioeconomic status and pattern of presence/absence of psychosocial problems)

Items	No problems (*n* = 1812)	Problems at age 2 (*n* = 296)	Problems at age 3 (*n* = 195)	Continuing problems (*n* = 206)
		Adjusted model	Adjusted model	Adjusted model
	OR (95% CI)	OR (95% CI)	OR (95% CI)	OR (95% CI)
Maternal education level				
Low vs. high	Reference	2.21** (1.48–3.29)	1.72* (1.06–2.79)	3.90** (2.49–6.13)
Middle vs. high	Reference	1.41* (1.06–1.87)	1.12 (0.79–1.58)	2.14** (1.50–3.07)
Single-parent family				
One-parent vs. two-parent	Reference	1.25 (0.74–2.11)	1.21 (0.64–2.27)	1.82* (1.10–3.01)
Unemployment in the family				
Yes vs. no	Reference	1.38 (0.89–2.15)	0.83 (0.46–1.51)	1.99* (1.23–3.21)
Financial problem in the family				
Yes vs. no	Reference	1.26 (0.70–2.28)	2.14* (1.11–4.14)	1.50 (0.82–2.77)
Neighborhood socioeconomic status				
Low vs. high	Reference	1.02 (0.76–1.37)	1.08 (0.77–1.52)	1.32 (0.91–1.92)
Middle vs. high	Reference	0.92 (0.63–1.34)	0.75 (0.46–1.21)	1.04 (0.63–1.71)

This table presents imputed data

Adjusted model: the model additionally adjusted for child gender and child ethnic background. ‘No problems’ group is the reference group of patterns of presence/absence of psychosocial problems

*OR* odds ratio, *CI* confidence interval;

*p < 0.05; **p < 0.001

Children from mothers with a low education level, compared to those from a mother with a high education level, had higher odds to be in the the ‘problems at age two’ group (OR = 2.21, 95% CI 1.48–3.29), be in ‘problems at age three’ group (OR = 1.72, 95% CI 1.06–2.79), and be in the ‘continuing problems’ group (OR = 3.90, 95% CI 2.49–6.13). Children from a mother with a middle education level, compared to those from a mother with a high education level, had higher odds to be in the ‘problems at age two’ (OR = 1.41, 95% CI 1.06–1.87) and be in the ‘continuing problems’ group (OR = 2.14, 95% CI 1.50–3.07).

Children from single-parent families, compared to those from two-parent families, had higher odds to be in the ‘continuing problems’ group (OR = 1.82, 95% CI 1.10–3.01). For the economic stress events before children age 2 years, children from family experienced unemployment (OR = 1.99, 95% CI 1.23–3.21) had higher odds to be in the ‘continuing problems’ group; children from family experienced financial problems (OR = 2.14, 95% CI 1.11–4.14) had higher odds to be in the ‘problems at age three’ group, compared to those from family without this experience. Neighborhood SES was not associated with the patterns of presence/absence of psychosocial problems in the regression model.

### Additional analyses

Compared to participants only participating at age 2 (*n* = 775), children and parents in the assessment at age 3 (*n* = 2734) were more likely to have a Dutch ethnic background and a higher education level for parents (all *p* < 0.001). No significant differences were found between boys and girls (*p* > 0.05) (Supplementary Table S2). Supplementary Table S3 shows the results of multivariate logistic regression conducted with complete data. The results regarding the full models of multivariate logistic regression conducted with non-imputed data and those with imputed data were similar.

## Discussion

This study examined the longitudinal association between SES and the pattern of presence/absence of psychosocial problems in early childhood. A lower SES, as indicated by maternal education level, single-parent family, unemployment, and financial problems in the family, was associated with higher odds for continuously having psychosocial problems in children age 2–3 years. In addition, children from lower educated mothers and children from families who experienced financial stress had higher odds of presenting psychosocial problems at age 2 or 3 years. Neighbourhood SES was not associated with the pattern of presence/absence of psychosocial problems in this study.

In the first set of analyses, we examined proportions of preschool children who had psychosocial problems at age 2 and age 3 separately. Around one-fifth (2*Y* = 20.0%, 3*Y* = 16.0%) of all children met the BITSEA criteria of psychosocial problems. The rates were closed to 13.8% found in 3–4-year-old children using Strengths and Difficulties Questionnaire (SDQ) and 18.4% found in preschool children using Diagnostic and Statistical Manual, third edition (DSM III-R) [31]. Next, four groups of pattern of presence/absence of psychosocial problems were created based on the two assessments at age 2 and 3. As expected, children without psychosocial problems at both ages formed the largest group (72.2%). Concerning disappearance of children’s psychosocial problems, half of the children with psychosocial problems at age 2 did not have psychosocial problems at age 3. Previous studies reported similar change but lower rates of children back to normal development from risky status during time [22, 32, 33]. However, populations of these studies were school-age children and adolescents. More studies in this field on young children are still needed. Poor psychosocial health in early childhood may affect later well-being in a 16-year follow-up study [1]. Therefore, it is important to better understand the psychosocial problems in young children and these changes in psychosocial problems.

In the second set of analyses, when comparing the ‘no problems’ group (as reference) and other three groups in multinomial logistic regression analyses, lower education levels of mothers were associated with higher odds of having problems at age two, having problems at age 3, and having problems at age 2 and 3. Especially, children from low-educated mothers increased by almost three times odds of belonging ‘continuing problems’ group. The study by Kuruczova et al. reported a similar longitudinal association between lower maternal education levels and children’s psychosocial problems at age 7, 11, 15, and 18 years [32]. Educational attainment is the most important determinant of health literacy, low-educated mothers may lack enough health literacy to recognize children’s problems and have more healthcare access [34]. However, the exact mechanisms underlying the maternal education level and change in psychosocial problems in early childhood are unclear.

Also, living in a single-parent family was shown to result in higher odds of being in the ‘continuing problems’ group. This finding was consistent with findings that children from single-parent families had higher scores for all SDQ subscales than children from two-parent families in the 11-year follow-up study [32]. It was suggested that two-parent families, also if there is a ‘new partner’ families, may relief some of the psychosocial, financial, and relational burdens associated with single-parent families [32, 35].

Unlike previous studies, we also examined whether financial stress in the family (i.e., unemployment and financial problems) were associated with the pattern. It is worth noting that unemployment and financial problems in the family after childbirth did increase the odds of belonging to ‘continuing problems’ group and ‘problems at age three’ group, respectively, for children age 2–3 years. It was suggested that the financial stress might impact child’s health outcomes directly through material deprivation and indirectly through parenting [36]. Our findings highlight the potentially longitudinal impact of financial stress events in early childhood on psychosocial health, even in preschool children. Research might take financial stress in early childhood into consideration when further exploring associations and mechanisms between SES and psychosocial development in children.

Findings of recent research in neighborhood SES and children’s psychosocial health were contradictory [17, 37]. The review study by Poulain et al. reported the importance of neighborhood SES as an area-level measurement in children’s psychosocial health [37]. However, Boelens et al. reported no associations were found in 4–12-year-old children [17]. The non-significant associations in the present study might result from the young population and the single neighborhood indicator in this study. First, children ages 2–3 years might be less influenced by neighborhood environment compared to domestic environment due to the limited social interaction. Second, although neighborhood SES in this study was a comprehensive indicator with regard to adult residents’ income, education level and employment in the neighborhood, the neighborhood SES is a combination of several sociodemographic factors. Multidimensional indicators to evaluate neighborhood SES are still needed in future research.

In sum, this study examined the psychosocial problems and pattern of presence/absence of psychosocial problems in children age 2–3 years. Associations between lower SES and higher risk of psychosocial problems development found in middle childhood (9–13 years) [33, 38] were confirmed in this study. Since SES at different points in a child’s lifetime may have different impact on health outcomes, this study added to the existing literature by focusing on early childhood [39]. Especially, lower education levels of mothers were associated with higher odds of psychosocial problems in children at either age 2, age 3, or both ages. Previous studies reported that high maternal education could contribute to a faster decline in problems over time and weaken the relationship between SES and health [32, 40]. In our study, two measurements could not describe reliable trajectories of psychosocial development. Moreover, since psychosocial development is a time-long process, the 1-year follow-up was too short to measure the full change in early childhood. Future studies are recommended to conduct long-term follow-up periods combining multi-measurements in early childhood, taking into account the neighborhood SES and financial stress to make comprehensive conclusions.

### Implications for practice and future research

Our study findings support an association between SES and psychosocial problems in young children. The findings on the association between neighborhood SES and psychosocial problems in this study are, however, inconsistent with a previous study in school-aged children in the Netherlands [17]. The SES and health relation across the lifespan could be dynamic and vary in strength across different life stages [21, 41]. Youth health care professionals and researchers should take these age-specific characteristics into consideration. Moreover, youth health care professionals may pay a specific attention to the trajectory of psychosocial health development. Although the developmental stage accounts for patterns of presence and absence of psychosocial problems, the findings indicate a subgroup of children with continuing problems. Especially for this subgroup, personalized care might benefit them to prevent long-term mental health problems at later ages [42]. More longitudinal studies are needed to follow up with children over time, collecting information on psychosocial well-being, care use, and SES. Furthermore, health literacy has been suggested as a mediator in the association between SES and psychosocial development [34]. This finding is important for youth professionals and policymakers, as the information they provide needs to be tailored to families and children in order to reach them and provide adequate support.

### Methodological considerations

Findings in the present paper need to be viewed in light of some limitations. First, the measurement of psychosocial problems, BITSEA, was parent-reported. Therefore, parents may have under- or overestimated their child’s socio-emotional development. However, BITSEA is still a validated and reliable measurement with high internal consistency and validity with Child Behavior Checklist (CBCL), the clinical diagnosis instrument of children’s psychosocial problems [24]. Second, the average follow-up duration was 1 year, while changes in psychosocial problems among children are likely to happen over a longer period. However, this study focused on the change of presence/absence of psychosocial problems in early childhood. Future research is recommended to study the onset of children’s psychosocial problems in preschool children timely and assess the long-term effect in later years. Third, data on family income were missing in the present study; therefore, financial stress events (unemployment and financial problems) were used as the indicators of family SES. Although financial stress events might be considered a measure related to SES, it might be a consequence of unemployment that can occur in high- and low-SES families [4]. Moreover, Chen et al. suggested that accumulation of socioeconomic status in terms of family income across childhood is more important than social mobility or variability in socioeconomic status [39]. Therefore, family income, as an objective and direct measure of SES, is recommended to use in the future SES studies. Fourth, multicollinearity for the indicators of SES has been examined and dealt according to the Spearman coefficient. Paternal education level was excluded due to high correlation with maternal education level. However, multicollinearity resulting from low correlated indicators of SES could still exist in the present study. For example, family that experienced unemployment could be more possible to have financial problems. Therefore, not only adding the family income, SES based on a set of objective and valid indicators is needed in future study. Fifth, although the time between data collection waves was short (i.e., 1 year), family SES and neighborhood SES measured at baseline could have changed. Findings from a national birth cohort in the Netherlands indicated that social mobility through parent education was associated with children’s physical health [43]. Therefore, we recommend future studies to study potential changes in SES, social mobility, and psychosocial health outcomes. Sixth, evidence from similar studies conducted in developed countries with universal health care was used to compare and discuss with findings in the present study. However, SES might affect psychosocial based on the sociopolitical, cultural, or normative composition of a given country (e.g., access to free healthcare, family's experiences of poverty-related stress, and ethnic background) [11, 12, 22, 44]. Seventh, children at risk of psychosocial problems in this study included children who were at risk on the Problem scale, Competence scale, or both scales of the BITSEA. These children might be different subgroups. Future studies might differentiate children with different risk when the population for analyses is large enough to examine the difference between subgroups. Eighth, the parents with a Dutch ethnic background and a higher education level were more likely to participate in the follow-up of the study. Consequently, the findings are applicable to the population under study. Regardless, efforts should be made to involve hard-to-reach populations in research studies. Ninth, both cohorts included recruited similar community population and took similar measures of independent and dependent variables, making it possible to combine these datasets. There were differences between demographic characteristics of two cohorts, resulting in the combined population a better representativeness of 2-year-old children and their parents in this area. Moreover, sensitivity analyses showed no indication of differences between cohorts with regard to the association between indicators of SES and psychosocial problems. However, there might still be unobserved effect of combining data of two cohorts. Future studies could avoid such limitation by enrolling a larger sample population in the beginning. Finally, although early parenting explained a certain degree of socioeconomic gradient in psychosocial problems of children, much of the variance is still unexplained [45]. Further research may be called to reveal how this socioeconomic gradient is fully explained.

## Conclusion

Results suggest children in a lower SES, indicated by maternal education, single-parent family, and financial stress, had higher odds of developing and continuously having psychosocial problems in early childhood. These findings call for optimally timing interventions to reduce the impact of disadvantaged SES in early childhood on psychosocial health.

### Supplementary Information

Below is the link to the electronic supplementary material.

Supplementary file1 (DOCX 34 KB)

## Data Availability

Data are available upon request by contacting the corresponding author.
