# Supplementary material for: Longitudinal associations between socioeconomic status and psychosocial problems in preschool children
Source: Eur Child Adolesc Psychiatry. 2023 May 17;33(4):1029–38. doi: 10.1007/s00787-023-02217-5 (PMC11032269; doi:10.1007/s00787-023-02217-5)
Supplement: Supplementary file 1 — Supplementary file1 (DOCX 34 KB) [file 787_2023_2217_MOESM1_ESM.docx]

## Supplementary materials

Parents visited YHC and complete 24-month questionnaire

(n=3499)

parents included in the analyses

(n=2509)

Lost to follow-up: 765

Figure 1. Population of Analysis

Excluded for analyses:

-The second child of twins (n=26)

-Follow-up questionnaire completed by other caregiver than parents (n=32)

-Child age exceeded the age limitation of BITSEA (n=60)

-Missing in the BITSEA (n=64)

parents of 3-year-old children completed the follow-up questionnaires

(n=2734)

Supplementary Table 1. Multivariable logistic regression models (family socioeconomic status at age two and prevalence of psychosocial problems at age two and three)

| Items | Psychosocial problems at age two | |  | Psychosocial problems at age three | |
| --- | --- | --- | --- | --- | --- |
|  | Crude model | Adjusted model |  | Crude model | Adjusted model |
|  | OR (95%CI) | OR (95%CI) |  | OR (95%CI) | OR (95%CI) |
| Maternal education level |  |  |  |  |  |
| Low vs. High | 3.12** (1.98-4.92) | 2.63** (1.64-4.20) |  | 2.57** (1.58-4.19) | 2.51** (0153-4.13) |
| Middle vs. High | 1.60* (1.12-2.31) | 1.45 (1.00-2.10) |  | 1.35 (0.92-1.99) | 1.27 (0.85-1.89) |
| Single-parent family |  |  |  |  |  |
| Yes vs. No | 1.56 (0.89-2.73) | 1.46 (0.81-2.62) |  | 2.10* (1.20-3.65) | 2.16* (1.21-3.86) |
| Unemployment in the family |  |  |  |  |  |
| Yes vs. No | 1.29* (1.01-1.65) | 1.34*(1.04-1.72) |  | 1.49* (1.16-1.90) | 1.53* (1.19-1.97) |
| Financial problem in the family |  |  |  |  |  |
| Yes vs. No | 1.65**(1.25-2.18) | 1.56* (1.17-2.08) |  | 1.58* (1.18-2.11) | 1.46* (1.08-1.98) |
| Neighborhood socioeconomic status |  |  |  |  |  |
| Low vs. High | 1.20 (0.82-1.74) | 1.00 (0.68-1.48) |  | 1.16 (0.78-1.73) | 1.09 (0.72-1.65) |
| Middle vs. High | 1.01 (0.62-1.65) | 1.08 (0.65-1.77) |  | 0.98 (0.58-1.67) | 1.06 (0.62-1.81) |

Note: This table presents imputed data.

Model 1: The model with maternal education level, maternal work status, single-parent family, unemployment in the family, financial problems in the family, and neighborhood socioeconomic status as independent variables.

Model 2: The model additionally adjusted for child gender and child ethnic background.

Continued normal status is the reference category of change of social-emotional status.

Abbreviation: OR=odds ratio, CI=confidence interval;

* *p* <0.05; ** *p* <0.001.

Supplementary Table 2. Non-response analyses (n=3499)

|  | Total  (n=3499) | participants only at age two (n=775) | participants at age three  (n=2734) | *P*-value |
| --- | --- | --- | --- | --- |
| Child gender |  |  |  | 0.102 |
| Boy | 1745 (50.1) | 401 (52.7) | 1344 (49.3) |  |
| Girl | 1740 (49.9) | 360 (47.3) | 1380 (50.7) |  |
| Child ethnic background |  |  |  | <0.001 |
| Dutch | 2420 (72.3) | 415 (58.9) | 2005 (75.9) |  |
| Non-Dutch | 925 (27.7) | 289 (41.1) | 636 (24.1) |  |
| Maternal ethnic background |  |  |  | <0.001 |
| Dutch | 2261 (67.9) | 357 (50.6) | 1904 (72.6) |  |
| Non-Dutch | 1068 (32.1) | 348 (49.4) | 720 (27.4) |  |
| Paternal ethnic background |  |  |  | <0.001 |
| Dutch | 2320 (70.0) | 379 (54.9) | 1941 (74) |  |
| Non-Dutch | 992 (30.0) | 311 (45.1) | 681 (26) |  |
| Maternal education level |  |  |  | <0.001 |
| High | 1791 (53.7) | 281 (39.7) | 1510 (57.5) |  |
| Middle | 1160 (34.8) | 329 (46.5) | 831 (31.6) |  |
| Low | 385 (11.5) | 98 (13.8) | 287 (10.9) |  |
| Paternal education level |  |  |  | <0.001 |
| High | 1559 (48.6) | 244 (36.4) | 1315 (51.9) |  |
| Middle | 1164 (36.3) | 299 (44.6) | 865 (34.1) |  |
| Low | 484 (15.1) | 128 (19.1) | 356 (14) |  |

Note: Data presented as number (percentage).

*Missing item: Child-gender=14; Child-ethnic background=154; Mother-ethnic background=170; Father-ethnic background=187; Mother-education level=163; Father-education level=292;

*P* values are based on χ2 tests.

Supplementary Table 3. Multinomial logistic regression models in non-imputed complete data (n=2254)

| Items | No problems | Problems at age two | Problems at age two and three | Problems at age three |
| --- | --- | --- | --- | --- |
|  | OR (95%CI) | OR (95%CI) | OR (95%CI) | OR (95%CI) |
| Maternal education level |  |  |  |  |
| Low vs. High | Reference | 2.20** (1.42-3.40) | 4.28** (2.64-6.93) | 1.92* (1.15-3.22) |
| Middle vs. High | Reference | 1.44* (1.08-1.93) | 2.15**(1.48-3.13) | 1.12 (0.78-1.60) |
| Single-parent family |  |  |  |  |
| Yes vs. No | Reference | 1.26 (0.70-2.27) | 1.97* (1.11-3.48) | 1.43 (0.73-2.78) |
| Unemployment in the family |  |  |  |  |
| Yes vs. No | Reference | 1.33 (0.83-2.15) | 2.76** (1.70-4.49) | 0.72 (0.37-1.41) |
| Financial problems in the family |  |  |  |  |
| Yes vs. No | Reference | 1.51 (0.81-2.82) | 1.28 (0.66-2.45) | 2.55* (1.27-5.12) |
| Neighborhood socioeconomic status |  |  |  |  |
| Low vs. High | Reference | 0.95 (0.70-1.29) | 1.36 (0.91-2.03) | 1.08 (0.75-1.55) |
| Middle vs. High | Reference | 0.88 (0.59-1.31) | 1.09 (0.64-1.85) | 0.78 (0.47-1.30) |

Note: This table presents non-imputed complete data.

The missing numbers of variables are: Mother-education level (n=54); Mother-work status (n=259); Single-parent family (n=84); Unemployment (n=46); Financial problem (n=49); Neighborhood socioeconomic status (n=37).

Models with maternal education level, maternal work status, single-parent family, unemployment in the family, financial problems in the family, and neighborhood socioeconomic status as independent variables, and adjusted for child gender and ethnic background.

‘No problems’ group is the reference group of patterns of presence/absence of psychosocial problems.

Abbreviation: OR=odds ratio, CI=confidence interval;

* *p* <0.05; ** *p* <0.001.

Supplementary Table 4. sociodemographic characteristics of the populations from the two cohorts

|  | Total  (n=2509) | participants from cohort 1 (n=1063) | Participants from cohort 2  (n=1446) | *P*-value |
| --- | --- | --- | --- | --- |
| Child gender |  |  |  | 0.687 |
| Boy | 1232 (49.2) | 528 (49.7) | 704 (48.9) |  |
| Girl | 1272 (50.8) | 535 (50.3) | 737 (51.1) |  |
| Child ethnic background |  |  |  | <0.001 |
| Dutch | 1886 (76.9) | 764 (71.9) | 1122 (80.7) |  |
| Non-Dutch | 568 (23.1) | 299 (28.1) | 269 (19.3) |  |
| Maternal education level |  |  |  | <0.001 |
| High | 1412 (57.8) | 566 (55.2) | 846 (59.6) |  |
| Middle | 780 (31.9) | 297 (28.9) | 483 (34.0) |  |
| Low | 253 (10.3) | 163 (15.9) | 90 (6.3) |  |
| Paternal education level |  |  |  | <0.001 |
| High | 1231 (52.1) | 506 (51.8) | 725 (52.3) |  |
| Middle | 810 (34.3) | 303 (31.0) | 507 (36.6) |  |
| Low | 322 (13.6) | 167 (17.1) | 155 (11.2) |  |
| Single family |  |  |  | 0.877 |
| No | 2289 (94.4) | 951 (94.4) | 1338 (94.3) |  |
| Yes | 137 (5.6) | 56 (5.6) | 81 (5.7) |  |

Note: Data presented as number (percentage).

*Missing item: Child-gender=5; Child-ethnic background=55; Mother-education level=64; Father-education level=146; Single-parent family=83.

*P* values are based on χ2 tests.

Supplementary Table 5. Multinomial logistic regression models adjusted by cohort variable

| Items | No problems (n=1812) |  | Problems at age two (n=296) |  | Problems at age three (n=195) | Continuing problems (n=206) |
| --- | --- | --- | --- | --- | --- | --- |
|  |  |  | Adjusted model |  | Adjusted model | Adjusted model |
|  | OR (95%CI) |  | OR (95%CI) |  | OR (95%CI) | OR (95%CI) |
| Maternal education level |  |  |  |  |  |  |
| Low vs. High | Reference |  | 2.07** (1.38-3.09) |  | 1.67* (1.03-2.72) | 3.65** (2.32-5.76) |
| Middle vs. High | Reference |  | 1.43* (1.08-1.89) |  | 1.13 (0.80-1.59) | 2.17** (1.52-3.11) |
| Single-parent family |  |  |  |  |  |  |
| One-parent vs. two-parent | Reference |  | 1.30 (0.76-2.10) |  | 1.22 (0.65-2.30) | 1.89* (1.14-3.14) |
| Unemployment in the family |  |  |  |  |  |  |
| Yes vs. No | Reference |  | 1.45 (0.93-2.26) |  | 0.85 (0.47-1.54) | 2.09* (1.29-3.38) |
| Financial problem in the family |  |  |  |  |  |  |
| Yes vs. No | Reference |  | 1.19 (0.66-2.17) |  | 2.11* (1.09-4.08) | 1.42 (0.77-2.63) |
| Neighborhood socioeconomic status |  |  |  |  |  |  |
| Low vs. High | Reference |  | 0.97 (0.72-1.30) |  | 1.05 (0.74-1.49) | 1.25 (0.86-1.83) |
| Middle vs. High | Reference |  | 0.90 (0.61-1.31) |  | 0.74 (0.46-1.20) | 1.02 (0.62-1.69) |

Note: This table presents imputed data.

Adjusted model: The model additionally adjusted for child gender, child ethnic background, and original cohort.

‘No problems’ group is the reference group of patterns of presence/absence of psychosocial problems.

Abbreviation: OR=odds ratio, CI=confidence interval;

* *p* <0.05; ** *p* <0.001.
